# *Cadm1* Is a Metastasis Susceptibility Gene That Suppresses Metastasis by Modifying Tumor Interaction with the Cell-Mediated Immunity

**DOI:** 10.1371/journal.pgen.1002926

**Published:** 2012-09-20

**Authors:** Farhoud Faraji, Yanli Pang, Renard C. Walker, Rosan Nieves Borges, Li Yang, Kent W. Hunter

**Affiliations:** 1 Metastasis Susceptibility Section, Laboratory of Cancer Biology and Genetics, National Cancer Institute, National Institutes of Health, Bethesda, Maryland, United States of America; 2 School of Medicine, Saint Louis University, St. Louis, Missouri, United States of America; 3 Howard Hughes Medical Institute–National Institutes of Health Research Scholars Program, Chevy Chase, Maryland, United States of America; 4 Tumor Microenvironment Section, Laboratory of Cancer Biology and Genetics, National Cancer Institute, National Institutes of Health, Bethesda, Maryland, United States of America; Cincinnati Children's Hospital Medical Center, United States of America

## Abstract

Metastasis is a complex process utilizing both tumor-cell-autonomous properties and host-derived factors, including cellular immunity. We have previously shown that germline polymorphisms can modify tumor cell metastatic capabilities through cell-autonomous mechanisms. However, how metastasis susceptibility genes interact with the tumor stroma is incompletely understood. Here, we employ a complex genetic screen to identify *Cadm1* as a novel modifier of metastasis. We demonstrate that *Cadm1* can specifically suppress metastasis without affecting primary tumor growth. Unexpectedly, *Cadm1* did not alter tumor-cell-autonomous properties such as proliferation or invasion, but required the host's adaptive immune system to affect metastasis. The metastasis-suppressing effect of *Cadm1* was lost in mice lacking T cell–mediated immunity, which was partially phenocopied by depleting CD8^+^ T cells in immune-competent mice. Our data show a novel function for *Cadm1* in suppressing metastasis by sensitizing tumor cells to immune surveillance mechanisms, and this is the first report of a heritable metastasis susceptibility gene engaging tumor non-autonomous factors.

## Author Summary

Metastasis, the dissemination and growth of tumor cells in organs distinct from which they originated, is the most common cause of cancer-related death. Accumulating evidence indicates that an individual's genetic background, the heritable complement of genetic variations that distinguish individuals, not only contributes to overall cancer risk, but also specifically influences metastatic potential. Using a mouse model of metastatic breast cancer and complex genetic analysis, we have identified *Cadm1* as a metastasis susceptibility gene. *Cadm1* was previously identified as a tumor suppressor in lung adenocarcinoma, and reductions in its expression have been associated with poor survival in numerous cancer types. In this manuscript, we use *in vivo* modeling to show that high expression of *Cadm1* inhibits pulmonary metastasis, while knockdown of *Cadm1* promotes the metastatic capability of tumor cells. We further show that the metastasis-suppressive effect of *Cadm1* expression is lost in mice lacking T cell–mediated immunity and that this effect is partially mediated by CD8^+^ T-lymphocytes. Our data suggest that the inverse correlation between *Cadm1* expression and disease-free survival in humans is a result of a metastasis-suppressive interaction of *Cadm1* with the cell-mediated immunity.

## Introduction

Metastatic disease remains a major problem for the clinical treatment of many different malignancies. Metastases can appear years after treatment of the primary tumor and is frequently refractory to therapy [1]. It has been estimated that approximately 90% of cancer-related deaths are directly attributable to the development of metastatic disease, rather than the primary tumor [2]. In order for a tumor cell to form a clinically-relevant metastatic lesion, it must undergo a highly complex process termed the invasion-metastasis cascade, which includes escaping from the primary tumor, entering the circulation, evading the immune system, seeding the secondary organ, and adapting to growth in this foreign environment [3]. Evidence suggests that the invasion-metastasis cascade is driven by a complex interplay of tumor cell-autonomous properties and host derived factors [3]. There is also accumulating evidence that germline polymorphism modifies tumor cell metastatic capability, indicating that heritable genetic variability can predetermine a tumor cell's propensity to metastasize [4]–[6]. In this study, we employ a complex genetics screen that exploits the differential heritable metastatic susceptibility observed among strains of inbred mice to identify tumor-autonomous expression of *Cadm1* as a germline modifier of metastatic susceptibility. We demonstrate that over-expression of *Cadm1* by as little as 1.5-fold can specifically suppress metastasis without any resultant difference in primary tumor growth.

In addition to tumor-autonomous cellular phenotypes, metastatic efficiency is also impacted by tumor non-autonomous, host-derived factors including the immune system [3]. However, mechanisms by which tumor cells interact with the immune system remain poorly understood. Here, we show that the metastasis suppressive effects of *Cadm1* are lost in mice lacking functional T cell–mediated immunity, an effect which is partially phenocopied by the depletion of CD8^+^ T cells in immune-competent mice, suggesting that *Cadm1* sensitizes tumor cells to immune-surveillance mechanisms by CD8^+^ T cells. Since differences in expression of *Cadm1* are inherited in mice, our data links the contribution of the genetic background in determining metastatic risk to the adaptive immune system, suggesting that individuals with higher levels of *Cadm1* expression may be more resistant to metastasis.

## Results

### Complex genetic screen identifies *Cadm1* as a candidate metastasis susceptibility gene

**Figure 1:**
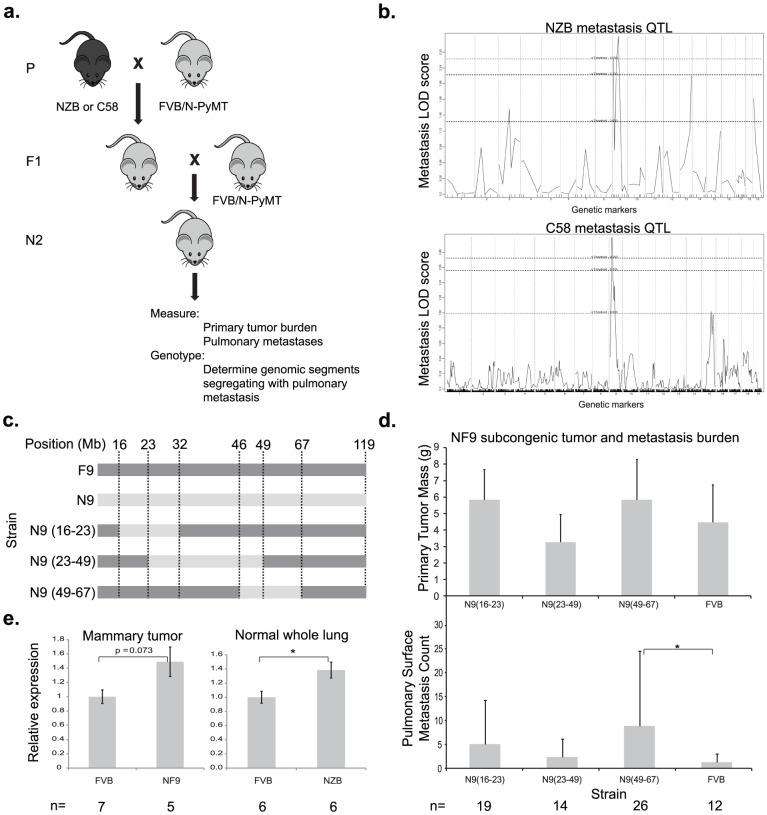
A genetic screen in mice identifies *Cadm1* as a candidate germline modifier of metastasis. (a) NZB and C58 mice were crossed to FVB mice then re-crossed with MMTV-PyMT-transgenic FVB mice. (b) Primary tumor, pulmonary metastasis, and genotype data was obtained from transgene-positive mice developed tumors and was used to generate QTL maps for pulmonary metastasis. Subcongenic lines containing fragments of NZB chromosome 9 (c) were crossed to MMTV-PyMT transgenic FVB mice and tumor size and pulmonary surface metastases were measured in the progeny (d). No significant change in tumor mass was observed, however a significant increase in metastasis was observed in the N9(49–60) subcongenic line (p = 0.022), resolving the locus of susceptibility was resolved to 49–60 Mb of chromosome 9. (e) Real time quantitative PCR measurement of cDNA from mammary tumor and lung tissue demonstrated a 1.5 fold higher expression of *Cadm1* in NZB and NF9 relative to FVB.

Previous work from our laboratory demonstrated that the progeny of FVB-MMTV-PyMT, a mouse model of metastatic breast cancer, and NZB/B1NJ or C58/J mice have significantly reduced pulmonary metastasis relative to the parental FVB-MMTV-PyMT [7]. Preliminary genetic mapping in an NZBxFVB backcross (Figure 1A) suggested the presence of a metastasis susceptibility gene on chromosome 9 [8] which was subsequently validated by the development of a chromosomal substitution strain [9]. Reproducible association of a metastasis susceptibility locus on proximal chromosome 9 was obtained by analysis of a 226 animal C58 x FVB backcross. Linkage analysis of the C58 backcross replicated the association with metastasis susceptibility for the proximal half of mouse chromosome 9 (Figure 1B and Figure S1). Despite the reduction of metastasis in the C58xPyMT or the NZBxPyMT F1 progeny, inheritance of either the C58 or NZB proximal chromosome 9 was associated with increased metastasis in the backcrosses. These data are consistent with the possibility that NZB and C58 harbor a common metastasis promoting allele whose presence was unmasked by segregation of unlinked compensatory metastasis-resistance genes in the backcross populations.

To further resolve the segment of chromosome 9 carrying this susceptibility locus, three congenic strains were developed that contained overlapping segments of NZB chromosome 9, spanning from 16–67 megabases (Figure 1C). Each congenic was bred to the PyMT transgenic animal and the effect of the NZB chromosomal segments on metastatic progression was assessed. No significant difference was observed for primary tumor burden. Only the congenic strain containing NZB chromosome 9 from 46–67 megabases was found to significantly increase metastatic susceptibility relative to FVB (p = 0.022, Figure 1D), consistent with the original quantitative trait locus (QTL) analysis. These results indicate that the gene(s) of interest resided within 46 Mb and 67 Mb of chromosome 9 and therefore subsequent analysis focused on this region.

To filter the gene list in the chromosome 9 metastasis modifier interval for potential candidate genes, haplotype analysis was performed. Based on the hypothesis that NZB and C58 shared a common metastasis susceptibility gene, screening for genes with identical haplotypes in NZB and C58 that were distinct from the FVB haplotype identified 80 potential candidate genes. Since the majority of polymorphisms occur in non-coding sequence it is hypothesized that the majority of quantitative traits are due to subtle changes in gene expression rather than non-synonymous amino acid substitutions. Based on this assumption the candidate gene list was further filtered by RNA expression microarray analysis. Tumors from PyMT-FVB or NZB chromosome 9 substitution (NF9) strains were subjected to microarray analysis to identify differentially expressed transcripts. Three genes within the candidate interval, *Cadm1*, *Zbtb16* and *Pias1*, had the appropriate haplotype structure and were differentially expressed between the two genotypes. Of the three *Cadm1* had the greatest magnitude of differential expression on microarray. Since aberrations in *Cadm1* expression have previously been associated with poor prognosis in numerous malignancies [10]–[14], *Cadm1* was selected for further characterization. *Zbtb16* and *Pias1* are under investigation in separate studies.

Exon sequencing of the *Cadm1* protein-coding exons was performed to confirm the predicted haplotype differences between NZB and FVB and to screen for undescribed polymorphisms. Sequence analysis confirmed that NZB and FVB have distinct haplotypes at the *Cadm1* locus and validated one synonymous polymorphism in exon 2 (rs32721609, Figure S2). Since no non-synonymous polymorphisms were observed, subsequent efforts were focused on the possible effect of expression levels on the metastatic phenotype.

To validate microarray data that *Cadm1* was differentially expressed in FVB and NZB, quantitative real time PCR (qRT-PCR) was conducted on reverse transcribed RNA extracted from PyMT-FVB and NF9 tumors. The results showed a trend toward increased *Cadm1* expression in NF9 tumors relative to FVB that was of borderline significance (p = 0.073, Figure 1E). To rule out the possibility that differential expression might have resulted from somatic alterations, expression was assessed in normal lung tissue as a representative epithelial sample. qRT-PCR in normal lung tissue from high-metastatic FVB and low-metastatic (NZB x FVB)F1 transgene-negative females showed a statistically significant increase in *Cadm1* expression in NZB F1 mice of about 1.5-fold (Figure 1E). These results, in combination with linkage and haplotype data, were consistent with the hypothesis that *Cadm1* expression is likely to be a germline modifier of metastasis.

### *Cadm1* expression inversely impacts metastasis

**Figure 2:**
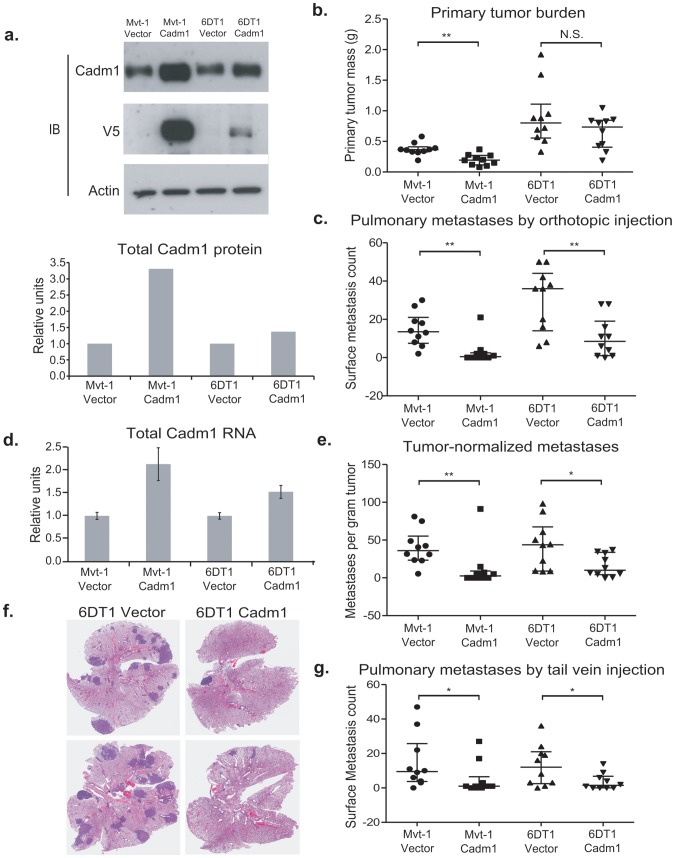
The effect of *Cadm1* over-expression on tumor growth and metastasis *in vivo*. (a) Expression of endogenous and exogenous V5-epitope tagged *Cadm1* protein (IB, immunoblot) and total protein by ECL densitometry on immunoblot. (d) Total RNA levels of *Cadm1* measured by qRT-PCR (mean +/− standard error of the mean [SEM]). Mvt-1 and 6DT1 mouse mammary tumor cells stably expressing *Cadm1* were implanted into mammary fat pads of syngeneic FVB mice. Data on primary tumor burden (Mvt-1: p = 0.0031; 6DT1: p = 0.2261) (b), pulmonary surface metastases (c) was collected on day 30 (Mvt-1: p = 0.0026; 6DT1: p = 0.0089; median +/− interquartile range; n = 10 mice per group). (e) Pulmonary surface metastases were normalized by primary tumor mass to determine if *Cadm1* overexpression showed an effect on metastasis independent of its potential tumor suppressive activity (Mvt-1: p = 0.0048; 6DT1: p = 0.0185; median +/− interquartile range; n = 10 mice per group). Arrows indicate several but not all visible metastatic nodules present on lung surfaces. (f) Images of whole lungs of two mice implanted with 6DT1 control cells and two mice implanted with 6DT1 cells expressing *Cadm1*. (g) Results of tail vein injection study (Mvt-1: p = 0.0361; 6DT1: p = 0.0475; median +/− interquartile range; n = 10 mice per group). (N.S., not significant; * defined as p<0.05; ** defined as p<0.01.)

*Cadm1*, also known as *Tslc1*, *Necl2*, *Ra175, IgSF4a and SynCAM* is an immunoglobulin superfamily adhesion molecule reported to be involved in homotypic and heterotypic cell-cell interactions [15]. It has also been identified as a tumor suppressor in lung adenocarcinoma [16]. To test whether variation in *Cadm1* expression had an impact on tumor growth and metastasis, two independent mouse mammary tumor cell lines stably expressing *Cadm1* were generated. Mvt-1 and 6DT1 cells [17] were transduced with Pol2-driven empty vector or *Cadm1* expression vector lentivirus particles and selected with blasticidin. Stable expression of exogenous *Cadm1* was confirmed by Western Blot and qRT-PCR (Figure 2A and 2D) and quantification revealed that 6DT1 cells over-expressed *Cadm1* by approximately 1.5 fold, similar to the difference observed in NF9 tumors and lung relative to FVB (Figure 1E and Figure 2D). One hundred thousand cells stably expressing *Cadm1* or empty vector were then injected into the fourth mammary fat pad of syngeneic mice. Thirty days post-injection mice were euthanized and primary tumors were resected and weighed to assess the effect of *Cadm1* on primary tumor growth. A statistically significant reduction in primary tumor growth was observed for the Mvt-1 cell line (Figure 2B). In contrast, no significant difference in primary tumor growth was observed 6DT1 cells.

The effect of ectopic *Cadm1* expression on metastasis was then assessed by pulmonary surface metastasis counts. To account for the reduction of primary tumor growth for the Mvt-1/*Cadm1* cell line, metastasis counts were also normalized by primary tumor burden to allow better comparisons between cell lines. As shown in Figure 2C, 2E, and 2F, *Cadm1* expression significantly reduced the metastatic capability of both Mvt-1 and 6DT1 relative to controls, consistent with a metastasis susceptibility role for *Cadm1*. The metastatic nodule size was measured on lung sections of orthotopically injected mice and did not differ significantly between control and *Cadm1* expressing cells (Figure S3). Significant reductions in pulmonary metastasis were also observed by intravenous injection of tumor cells into the tail-vein, a model that assays tumor cell colonization of the lung (Figure 2G), suggesting that the effect of *Cadm1* on metastasis operates downstream of local invasion and intravasation.

**Figure 3:**
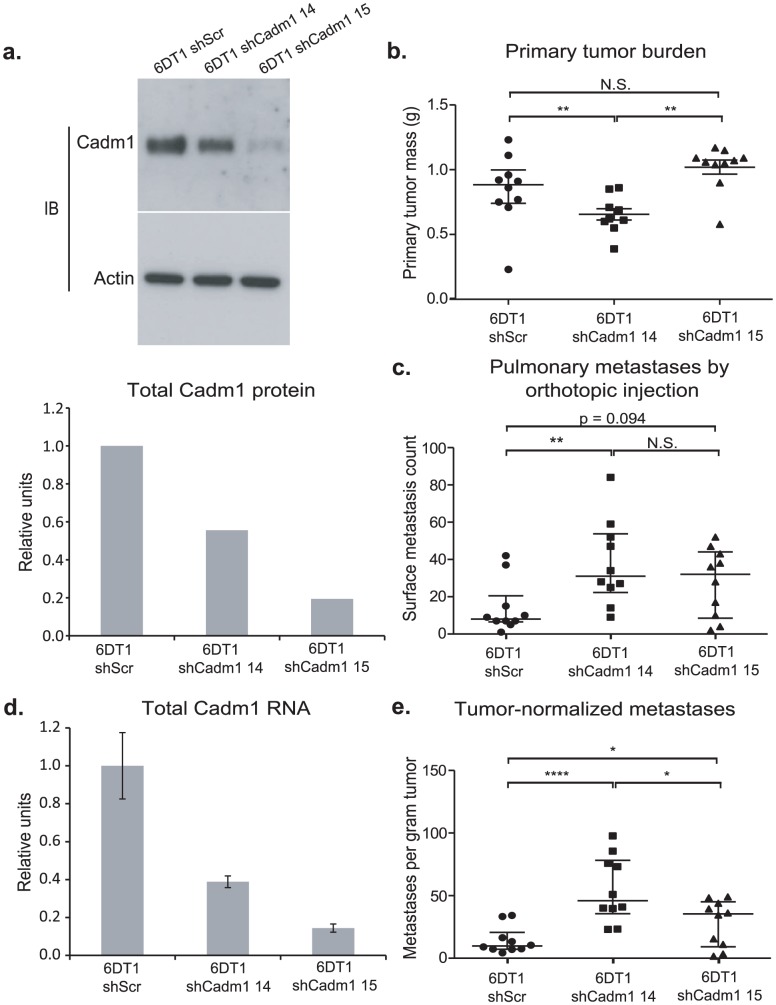
The effect of *Cadm1* knockdown on tumor growth and metastasis *in vivo*. 6DT1 cells expressing shRNA constructs targeting *Cadm1* showed (a) protein level knockdown of *Cadm1* by immunoblot and ECL densitometry quantification and (d) total RNA level knockdown (mean +/− SEM). Orthotopic implant data showing the effects of *Cadm1* knockdown on primary tumor burden (shScr vs. sh14: p = 0.0090; shScr vs. sh15: p = 0.0943; sh14 vs. sh15: p = 0.0001) (b), pulmonary surface metastases (shScr vs. sh14: p = 0.0085; shScr vs. sh15: p = 0.0709; sh14 vs. sh15: p = 0.3485) (c), and tumor-normalized metastases (e) ((shScr vs. sh14: p = 0.00005; shScr vs. sh15: p = 0.0444; sh14 vs. sh15: p = 0.0152; median +/− interquartile range; n = 10 mice per group).

Expression of *Cadm1* in 6DT1 cells suppressed metastasis without significantly impacting primary tumor growth, suggesting that, *Cadm1* might be functioning purely as a metastasis suppressor in this cell line. To test whether *Cadm1* had metastasis-specific effect on metastatic progression, knockdown of the endogenous gene in 6DT1 cells was performed as *Cadm1* expression in 6DT1 cells reproducibly has no effect on primary tumor growth. Due to the potentially confounding effect on the primary tumor the Mvt-1 cell line was excluded from this analysis. 6DT1 cells were transduced with two independent shRNA lentivirus constructs targeting *Cadm1* and were selected with puromycin, achieving 44% and 81% reduction of total *Cadm1* protein, respectively (Figure 3A and 3D). These cells were subsequently injected orthotopically into immune-competent syngeneic mice for *in vivo* analysis. Cells expressing shRNA-14 showed a statistically significant reduction in primary tumor mass while those expressing shRNA-15 showed no change in primary tumor mass (Figure 3B). The tumor suppression observed with shRNA-14 is likely to be an off-target effect since it was only observed with one of two shRNAs. Although only shRNA-14 significantly promoted metastasis (Figure 3C), both constructs showed significant increases in pulmonary metastasis after normalization for primary tumor burden (Figure 3E). Since increases in metastases were observed for both constructs *Cadm1* is likely to have a metastasis-specific function in this model system.

### *Cadm1* transgene expression is attenuated in metastatic nodules

**Figure 4:**
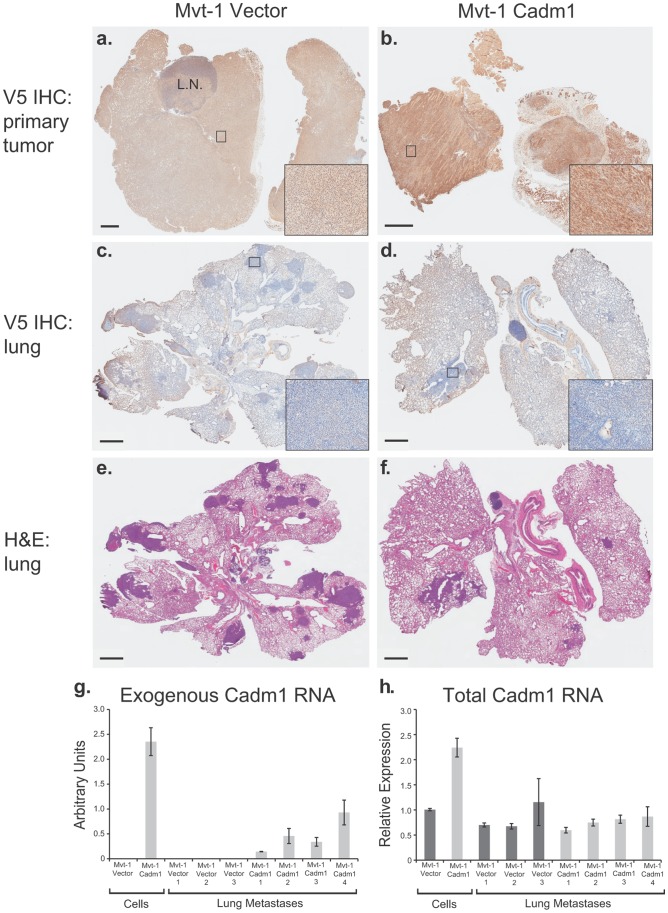
Pulmonary metastases of Mvt-1/*Cadm1* cells express attenuated levels of *Cadm1*. Matched primary tumor and lung sections were immunohistochemically (IHC) stained with antibody against the V5 epitope. Immunohistochemical staining of the primary tumors produced by control (a) and *Cadm1*-V5 (b) expressing cells. IHC stained lung sections of mice implanted with control (c) and *Cadm1*-V5 (d). Hematoxylin and eosin (H&E) stained lung sections of mice implanted with control (e) and *Cadm1*-V5 (f) expressing cells. Insets show 20× magnification of boxed area (L.N., lymph node; black bar indicates 1 um). Level of (g) exogenous and (h) total *Cadm1* mRNA from grossly dissected pulmonary metastatic nodules of mice injected with Mvt-1/Vector versus Mvt-1/*Cadm1* relative to the original cell lines prior to injection.

To assess if the reduction in metastasis observed in *Cadm1* over-expressing Mvt-1 cells was due to a tumor suppressor or metastasis-specific effect, immunohistochemistry was performed. Matched tumor and lung specimens were sectioned and staining was conducted against the V5 epitope to determine if pulmonary metastases from *Cadm1* positive tumors retained expression of V5-tagged *Cadm1*. As shown in Figure 4A–4F, the primary tumors (Figure 4A–4B) stained positive for the V5 epitope, however, pulmonary metastases (Figure 4C–4D) from matched tumors did not stain for V5, indicating that these metastases had lost expression of the *Cadm1* transgene.

Further, gross dissection with subsequent qRT-PCR of metastatic nodules from the lungs of mice injected intravenously with Mvt-1/*Cadm1* cells revealed that exogenous *Cadm1* mRNA expression was attenuated in all four mice relative to the levels expressed in those cells upon injection (Figure 4G) and total *Cadm1* expression was not significantly different between Mvt-1/Vector and Mvt-1/*Cadm1* bearing mice (Figure 4H). Since these cells were injected directly into the venous circulation and likely seeded the lungs immediately after injection, they had little time to lose *Cadm1* expression suggesting that tumor cells with lower overall *Cadm1* expression upon injection were selected for colonization. Together, this data suggests that low *Cadm1* expression may be required for successful lung colonization by Mvt-1 cells, consistent with a specific metastasis-suppressing role in this cell line.

### *Cadm1* expression does not affect cell-autonomous properties *in vitro*

Previous studies demonstrated that expression of *Cadm1* could affect *in vitro* phenotypes associated with metastatic potential [18]. To examine the mechanism by which *Cadm1* influences metastasis in breast cancer, proliferation, migration and invasion assays were performed. Ectopic expression of *Cadm1* had no significant impact on proliferation rates in either 2-D or 3-D culture, and no difference in trans-well migration or invasion (Figures S4, S5 and S6). Though *Cadm1*-expressing 6DT1 cells demonstrated a slight, marginally significant reduction in motility relative to control cells by scratch wound assay, Mvt-1 cells showed no difference in motility (Figure S7). These data would suggest that the effects of *Cadm1* are likely distinct from events leading to the initial escape from the primary tumor, which is supported by the observed suppression of metastasis upon tail vein injection (Figure 2G).

### *Cadm1* interacts with the cell-mediated immunity

**Figure 5:**
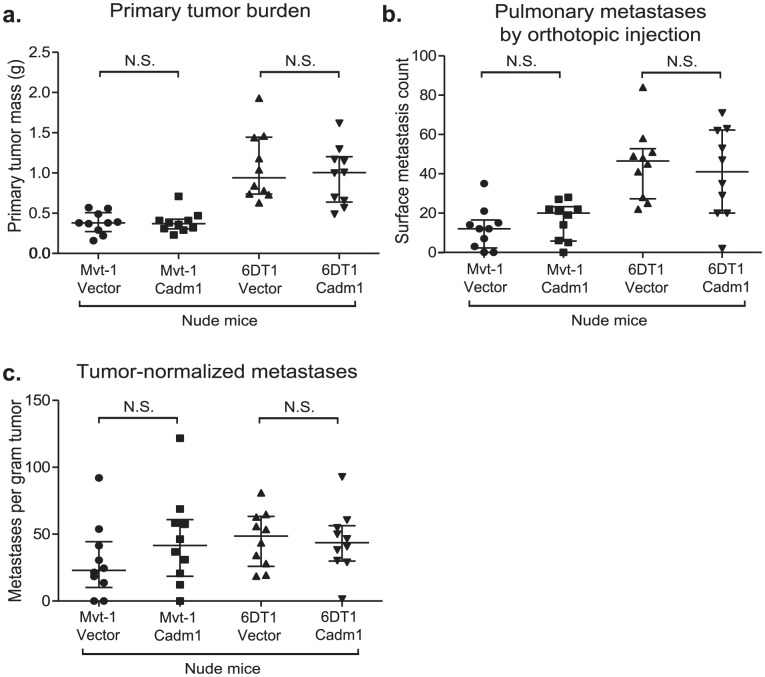
The effect of *Cadm1* expression on tumor and metastasis in athymic mice. Orthotopic injections of control and *Cadm1*-expressing cells into athymic nude mice resulted in no significant difference in (a) primary tumor burden, (b) pulmonary metastasis, or (c) tumor-normalized metastasis. (median +/− interquartile range; n = 10 mice per group).

*Cadm1* has been shown to engage in heterotypic interactions with class-I restricted T cell adhesion molecule (*Crtam*), a marker of activated CD8^+^ T cells and NK-cells [19]. This interaction has been shown to induce interferon-γ production by CD8^+^ T cells, enhance CD8^+^ T cell and NK-cell cytotoxicity *in vitro*, and tumor rejection *in vivo* [20], [21]. In order to test the role of cell-mediated immunity in *Cadm1*-mediated metastasis suppression, control and *Cadm1*-expressing cells were orthotopically implanted into athymic nude mice. As illustrated in Figure 5, the metastatic phenotype of *Cadm1*-expressing cells was rescued in immunocompromised mice. In addition, the tumor suppressive effect of *Cadm1* in Mvt-1 cells was also lost. This suggests that T cell mediated immunity may be critical for the tumor and metastasis suppressive effects of *Cadm1*.

**Figure 6:**
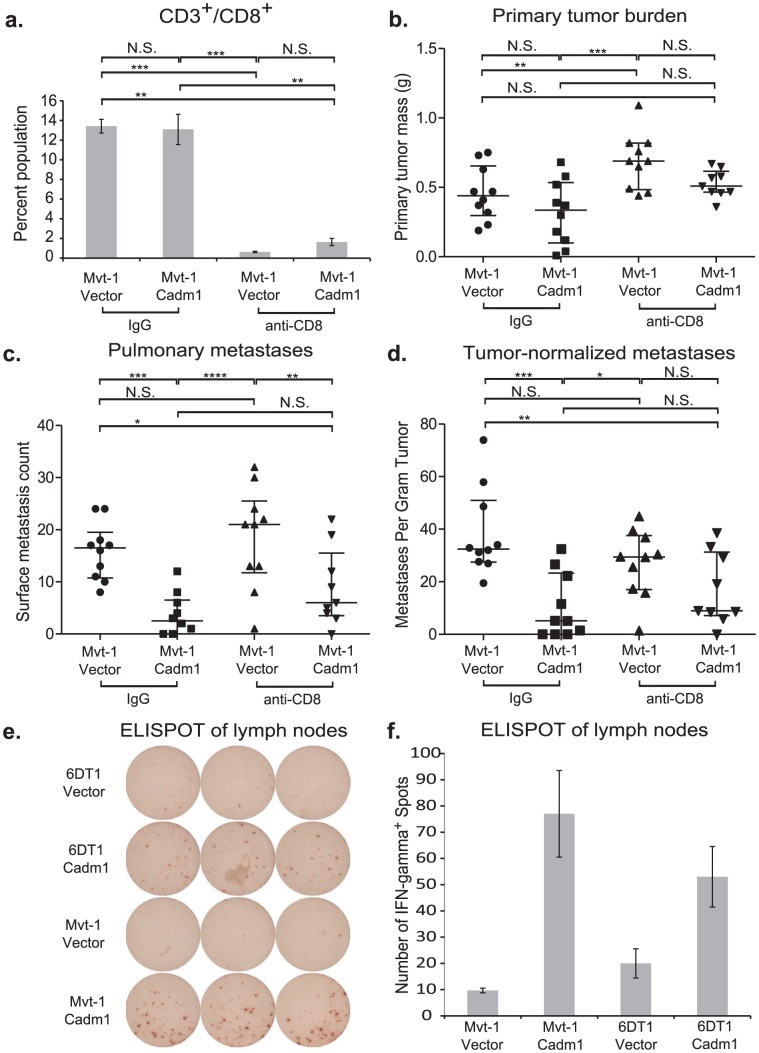
The effect of *Cadm1* expression on tumor and metastasis in CD8^+^ T cell depleted mice. Orthotopic injections of control and *Cadm1*-expressing Mvt-1 cells into immune-competent FVB mice that were treated with IgG or anti-CD8 resulted in (a) >90% depletion of CD8^+^ T cells. (b) No significant differences were observed in tumor burden between control and *Cadm1* expressing within the same antibody treatment group, though a significant increase was observed in control Mvt-1 cells upon anti-CD8 treatment. (c) Total pulmonary metastases showed significant suppression in Mvt-1/*Cadm1* in both control and anti-CD8 treatment groups. (d) Metastases normalized by matched primary tumor mass showed highly significant *Cadm1*-mediated metastasis suppression in the control IgG treatment group but was not significant in the anti-CD8 group (median +/− interquartile range; n = 10 mice per group). ELISPOT plate (e) and quantitative representation (f) of interferon-γ secreting T cells (mean +/− SEM). (N.S., not significant; * defined as p<0.05; ** defined as p<0.01; *** defined as p<0.001, **** defined as p<0.0001).

It has been reported that *Crtam* is present on activated natural killer (NK) and CD8^+^ T cells. Since athymic mice possess functional NK cells, we hypothesized that the loss of *Cadm1*-mediated metastasis suppression might be mediated by CD8^+^ T cells. Thus we orthotopically injected Mvt-1 cells expressing *Cadm1* in immune-competent mice in which CD8^+^ lymphocytes were depleted with anti-CD8 antibody (Figure 6A and Figure S8). Unexpectedly, *Cadm1*-expressing Mvt-1 did not suppress tumor growth in this experiment as previously observed (Figure 2C). However, subsequent protein-level expression analysis of the cells used in this experiment revealed that the level of *Cadm1* over-expression was lower than in previous studies (Figure S9) and similar to the levels observed in the 6DT1 cell line, suggesting that higher *Cadm1* expression is required to suppress primary tumor growth than to achieve metastasis suppression.

Although pulmonary metastases were significantly reduced in both control IgG treated and CD8^+^ lymphocyte-depleted mice, this effect was lost specifically in CD8^+^ lymphocyte-depleted mice when metastases were normalized by matched primary tumor mass (Figure 6B, 6C, and 6D). This suggests that CD8^+^ lymphocytes contribute to *Cadm1*-mediated metastasis and implicates the role of cell-mediated immunity in metastasis suppression by *Cadm1*.

The engagement of *Cadm1* on tumor cells and *Crtam* on CD8^+^ T cells has been reported to induce interferon-γ production of CD8^+^ T cells [20], [21]. We hypothesized that enhanced interferon-γ may mediate an inflammatory response to induce tumor cytotoxicity. To test whether interferon-γ secretion was enhanced in mice bearing *Cadm1*-expressing tumors relative to those bearing control tumors, we performed an ELISPOT assay. Cells derived from the draining lymph nodes contralateral to the tumor were used because the tumors frequently enveloped the ipsilateral lymph nodes. We observed a marked increase in interferon-γ secretion in lymphocytes derived from the lymph nodes of *Cadm1* ^+^ tumor bearing mice (Figure 6E and 6F), indicating that an interferon-γ-mediated immune response local to the primary tumor may be involved in *Cadm1*-mediated metastasis suppression.

### *Cadm1* expression predicts outcome in breast cancer patients

The down-regulation of *Cadm1* in invasive breast cancer, primarily by promoter hypermethylation, has been reported in at least four studies [22]–[25]. This loss or reduction in *Cadm1* expression is associated with increased tumor grade, stage, and local invasiveness [22]–[25]. To determine whether levels of *Cadm1* expression in breast tumors correlated with survival in patients, we searched three publicly available datasets from Oncomine, two of which have associated publications [26], [27] and the Gene expression-Based Outcome for Breast cancer Online (GOBO, http://co.bmc.lu.se/gobo/), a web-based meta-analysis tool containing microarray-based tumor expression data on 1881 patients from 11 public datasets [28].

**Figure 7:**
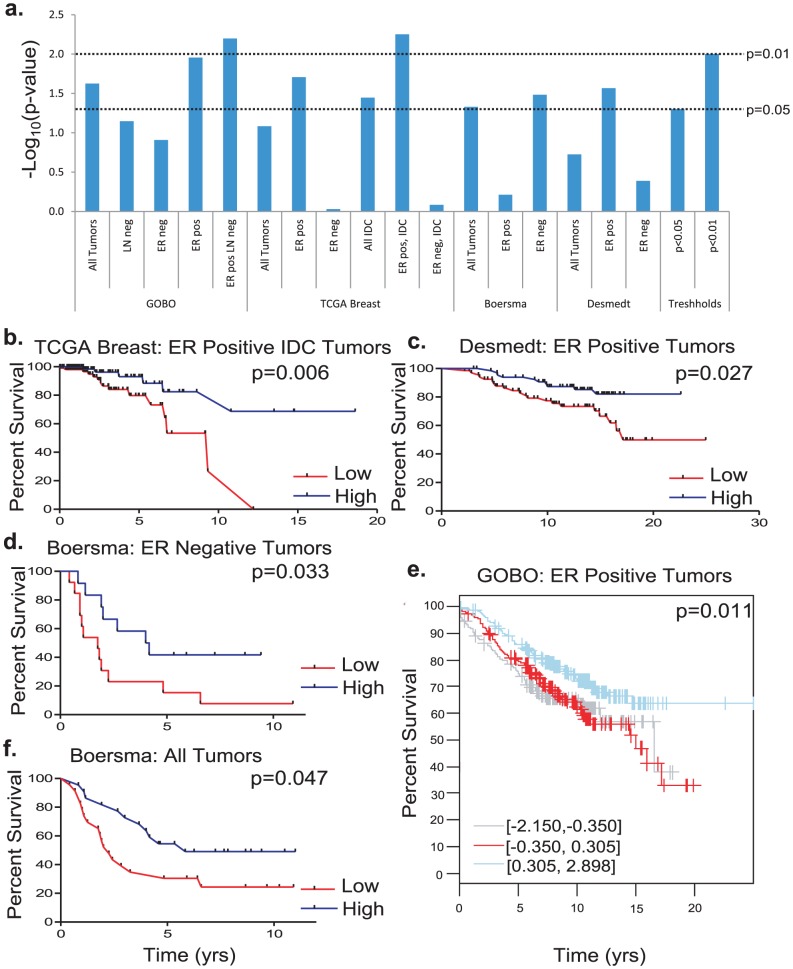
The effect of *Cadm1* expression levels on survival in patient datasets. (a) Compiled results of -Log_10_ transformed p-values for all survival analyses. Kaplan-Meier plots of (b) estrogen receptor (ER) positive invasive ductal carcinoma (IDC) tumors from the Oncomine TCGA set, (c) ER positive tumors from the Desmedt set, (d) ER negative tumors from the Boersma set, (e) ER positive tumors from GOBO, (f) all tumors from the Boersma set.

With the exception of the Boersma set, high *Cadm1* expression consistently correlated with significantly improved survival in estrogen receptor (ER) positive tumor datasets, (Figure 7A, 7B, 7C, 7E, and Table S1). In contrast, high *Cadm1* levels only significantly correlated with improved survival in ER negative patients in the Boersma dataset (Figure 7D and 7F), suggesting that any effect *Cadm1* levels may have on tumor progression are primarily relevant to ER positive tumors. Since metastasis is the primary determinant of overall survival in cancer, this suggests that high tumor cell *Cadm1* expression may also be protective against metastasis in human breast cancer.

## Discussion

Numerous studies have implicated down-regulation of *Cadm1* with poor patient prognosis [10], [12]–[14], [29], [30]; however, to the best of our knowledge no studies have investigated the mechanism responsible. Here we demonstrate that expression of *Cadm1* in two independent cell lines resulted in either specific metastasis suppression or tumor suppression accompanied by a reduction in metastasis. As metastasis is the primary cause of cancer-induced mortality, these findings imply a direct role for *Cadm1* in metastatic progression in breast cancer and provide a link between diminished *Cadm1* expression and reduced progression-free survival.

Two key observations suggest a direct role for *Cadm1* in metastasis suppression. First, in the Mvt-1 cell line, *Cadm1* ectopic expression was lost in all metastases, suggesting that down-regulation of *Cadm1* may be a prerequisite for completion of the metastatic cascade. Second, manipulation of *Cadm1* in 6DT1 cells specifically affected the ability to form pulmonary metastases, without any significant effect on primary tumor growth. Thus, in the 6DT1 cell line, *Cadm1* appears to function as a metastasis susceptibility gene, based on variations in functional levels. Together, the results suggest that *Cadm1* may have both tumor suppressive and metastasis suppressive activities which may depend on the level of *Cadm1* expressed.

The identification of *Cadm1* as an inherited metastasis suppressor gene was not predicted by the initial linkage analysis. Genetic analysis of both backcross populations and the congenic mouse strains indicated that inheritance of the C58 or NZB chromosome 9 candidate region was associated with an increase in pulmonary metastasis. In contrast, the up-regulation seen for both the C58 and NZB *Cadm1* alleles was associated with a decrease in pulmonary metastasis in the orthotopic transplant studies.

A likely explanation for this discrepancy lies with the complexity of the genome. Every individual carries numerous susceptibility and resistance factors and the final phenotype is dictated by the balance of these genes. The pro-metastatic chromosome 9 interval that was investigated in this study contains more than one gene that fit the criteria for a metastasis susceptibility gene. In addition, the strategy followed here does not capture candidates based on non-synonymous amino acid substitutions. We speculate the paradox between the identification of *Cadm1* as a metastasis-suppressing gene in a metastasis-promoting genomic region is explained by the presence of one or more metastasis-promoting genes within the interval. When inherited together the metastasis-promoting factors dominate, masking the effect of *Cadm1*.

Finally, our results potentially implicate *Cadm1* in cancer immunoediting, a process by which the adaptive immune system protects the host from developing cancer and, in turn, alters tumor progression by driving the outgrowth of tumor cells with decreased sensitivity to immune attack [31], [32]. We have shown that *Cadm1* may regulate metastasis by sensitizing tumor cells to surveillance by the immune system. Our findings show the metastatic potential of *Cadm1*-expressing cells was fully rescued in mice deficient of T cell mediated immunity, indicating that *Cadm1*-mediated metastasis suppression specifically involves the adaptive cell mediated immunity rather than the innate immunity, as the innate immune system is intact in athymic mice. However, we cannot rule out the possibility that thymus-dependent, *Cadm1*-sensitive immune cells are indirectly associated with metastasis suppression by recruiting innate immune cells, including NK-cells, to eliminate tumor cells. Since the effect of CD8^+^ T cells depletion did not result in a full rescue, we hypothesize that NKT cells, which also express the *Cadm1* receptor, *Crtam*, [33] and are absent in athymic mice [34] may also contribute to tumor cell cytotoxicity and clearance.

Further, the partial rescue of the metastatic phenotype by depletion of CD8^+^ T cells suggests that *Cadm1* may promote the detection of tumor cells by an immune surveillance mechanism which potentially results in the CD8^+^ T cell–mediated cytotoxicity and clearance of *Cadm1* expressing cells from circulation and secondary sites. While we have not directly shown that *Cadm1* expression resulted in tumor cell death by T cells, this notion is supported by previous studies [20], [21] showing *Cadm1* expression on target cells enhances T cell cytotoxicity in an MHC-dependent manner. Thus loss or reduction in *Cadm1* expression may contribute to the final step of cancer immunoediting: tumor cell “escape” from detection by the immune system [31]. Reports that the *Crtam-Cadm1* interaction induces interferon-γ secretion [20], [21] combined with our observation that lymph nodes draining *Cadm1^+^* tumors contain lymphocytes that secrete higher levels of interferon-γ than controls suggest that the interaction with the immune system may be mediated by engaging *Crtam* on cytotoxic lymphocytes. Further, the role of *Cadm1* as a tumor-autonomous component of immune surveillance is consistent with our observations that 1) knockdown of endogenous *Cadm1* potentiates metastatic capability, and 2) pulmonary metastases have lost expression of exogenous *Cadm1*. Together with the numerous [10]–[14] reports in the literature that *Cadm1* expression is lost in more invasive tumors, our data implicates *Cadm1* loss as an important step in cancer immunoediting that enhances the immune evasive and metastatic properties of tumor cells.

## Materials and Methods

### Mouse genetics (FVBxNZB backcross, generating and genotyping subcongenic mice, QTL analysis)

The NZB backcross has been described previously [8]. NZB chromosome 9 subcongenics were generated by breeding the NF9 chromosomal substitution line [9] to FVB/NJ, backcrossing the F1 progeny to FVB/NJ and screening the N2 backcross progeny for retention of NZB chromosomal segments. Chromosomal segments of interest were made homozygous by breeding to FVB/NJ followed by brother-sister mating of animals containing the segment of interest. Genotyping was performed by using a combination of microsatellite and SNP genotyping.

The C58 backcross was generated by crossing PyMT male animals with C58/J females to generate PyMT-positive F1 males, which were subsequently bred to FVB/NJ females to generate N2 backcross animals. Tumor phenotyping was performed as previously described [35]. Genotyping was performed using the Illumina Mouse Medium Density Linkage array at the Center for Inherited Disease Research (CIDR) using spleen DNA isolated by the Wizard DNA purification kit. Quantitative trait mapping (QTL) was performed using J/QTL [36].

### Exon sequencing of *Cadm1* from genomic DNA

Exons were amplified from genomic DNA then run on a 2% agarose gel. Amplified bands were purified by the Qiagen Gel Extraction Kit and cloned into TOPO-TA vectors (Invitrogen) and transformed into One-shot Top10 chemically competent E. Coli (Invitrogen) as per manufacture's protocol. Plasmid was purified using the Mini Prep kit (Qiagen). Sequencing reactions were conducted on ∼350 ng of purified plasmid using Big Dye Terminator reaction mix (Applied Biosystems) following the manufacturers protocol and sequenced by the NCI Sequencing Facility.

### Cell lines and culture conditions

All experiments were conducted using FVB/NJ background mouse mammary tumor explants cell lines 6DT1 and Mvt-1 kindly provided by Dr. Lalage Wakefield [17]. Cell lines were cultured in Dulbecco's Modified Eagle Medium (DMEM) (Gibco) supplemented with L-Glutamate (Gibco), 9% fetal bovine serum (FBS) (Gemini BioProducts), and 1% Penicillin and Streptomycin (P/S) (Gemini BioProducts).

### Oligonucleotides and plasmids

pDonr253 is a Gateway Donor vector modified from pDonr201 (Invitrogen). pDonr253 replaces the kanamycin resistance gene with a gene encoding spectinomycin resistance, and contains several sequencing primer sites to aid in sequence verification of Entry clones. The following oligonucleotides (Eurofins MWG Operon) were used in this study: L10379: 5′-GGGGACAACTTTGTACAAAAAAGTTGGCACCATGGCGAGTGCTGTGCTGCCGAGCGGATC

L10383: 5′-GGGGACAACTTTGTACAAGAAAGTTGAGATGAAGTACTCTTTCTTTTCTTC

The sequences of short hairpin RNA pLKO.1 vectors for *Cadm1* knockdown were

shRNA14: CCGGCGGACTGGTTTGTAAAGGAAACTCGAGTTTCCTTTACAAACCAGTCCGTTTTTG

shRNA15: CCGGCCTGTTCATCAATAACCTAAACTCGAGTTTAGGTTATTGATGAACAGGTTTTTG

### Cloning of *Cadm1*

Murine *Cadm1* was cloned into a Gateway Entry clone by PCR from a cDNA template (BC095986, RefSeq NM_018770.3). The Entry clone contains the complete ORF preceded by a Kozak translation initiation sequence and an ATG start codon, and lacking a stop codon at the 3′ end to allow C-terminal fusions to be generated. DNA was amplified using specific primers L10379 and 10383 containing the Gateway recombination sequences, and PCR was carried out using Pfusion polymerase (New England Biolabs). The final PCR product contains the gene of interest flanked on the 5′ side with a Gateway attB1 site and on the 3′ side with a Gateway attB2 site. The PCR product was cleaned using the QiaQuick PCR purification kit (Qiagen), and recombined into pDonr253 using the Gateway BP recombination reaction (Invitrogen) by the manufacturer's protocols. The subsequent Entry clone was sequence verified throughout the entire cloned region.

### Subcloning of *Cadm1* and control lentivectors

A lentiviral vector expressing a C-terminal V5 epitope-tagged fusion of *Cadm1* was generated using Multisite Gateway recombination. An entry clone using the murine Pol2 promoter was recombined with the *Cadm1* entry clone and a C-terminal entry clone encoding the V5 epitope tag (GKPIPNPLLGLDST) into a Gateway destination vector pDest-659. This vector is a modified version of the pFUGW lentiviral vector which contains the enhanced polypurine tract (PPT) and woodchuck regulatory element (WRE) to provide higher titer virus. In addition, it contains an antibiotic resistance gene for blasticidin resistance. Entry clones were subcloned by Gateway Multisite LR recombination using the manufacturer's protocols (Invitrogen). Expression clones were transformed into E. coli STBL3 cells to minimize unwanted LTR repeat recombination, and verified by agarose gel electrophoresis and restriction digest. Transfection-ready DNA for the final clones was prepared using the GenElute XP Maxiprep kit (Sigma). A control vector (8166-M24-658) was generated by standard Gateway LR recombination of a stuffer fragment made up of a non-coding DNA into the pLenti6-V5-DEST vector (Invitrogen). All lentivector constructs and lentivirus particles were generated by the Protein Expression Laboratory and the Viral Technology Group in NCI, Frederick.

### Viral transduction

Two milliliter suspensions of 5×10^4^ cells were incubated at 37°C in 5% CO_2_ overnight. Cells were then infected with 50 uL of concentrated lentivirus suspension, and selected 30 hours post-infection with 5 mg/mL blasticidin for over-expression (Invitrogen) constructs or 10 ug/mL (shRNA) puromycin for shRNA constructs.

### Immunoblot and antibodies

Protein was extracted by cell lysis in 400 uL of Pierce lysis buffer, vigorous homogenization, and incubation on ice for one hour. Twenty micrograms of protein extract per sample in NuPage LDS Sample Buffer and NuPage Reducing Agent (Invitrogen) were used for western blotting. PVDF membrane (Millipore) containing transferred proteins was incubated overnight with the primary antibodies: mouse anti-V5 (Invitrogen), chicken IgY anti-SynCAM/TSLC1/Cadm1 (MBL International), or mouse anti-β-actin (Abcam). The membrane was then incubated with horse-radish peroxidase linked anti-mouse (GE Healthcare) or anti-chicken IgY secondary antibodies (Abcam). Immunoblot was visualized using Amersham ECL Prime Western Blotting Detection System and Amersham Hyperfilm ECL (GE Healthcare). Densitometry data were obtained and analyzed with a ChemiDoc-It Imaging System and VisionWorksLS software (UVP).

### RNA isolation, reverse transcription, and real-time polymerase chain reaction (qRT-PCR)

RNA was isolated from tumors and cell lines using RNeasy kit (Qiagen) and reverse transcribed using iScript (Bio-Rad). Real-Time PCR was conducted using QuantiTect SYBR Green PCR kit (Qiagen). See Table S2 for primer sequences.

### Scratch wound assay

One-milliliter suspensions of 6×10^5^ cells were seeded in triplicate into each well of 24-well Essen ImageLock (Essen Bioscience) plates in selective media and incubated at 37°C and 5% CO_2_ overnight. The following day, the cells washed twice with PBS and incubated at 37°C and 5% CO_2_ for 3 hours in 10 ug/mL mitomycin C. Scratch wounds were made using Essen 4-channel scratch instrument loaded with Eppendorf 10 uL micropipette tips. Cells were subsequently washed three times with PBS, placed in selective media and placed in Incucyte (Essen Bioscience). Incucyte was programmed to image each well at 2-hour intervals. Data analysis was conducted using Incucyte 2011A software.

### Proliferation assay

One-milliliter suspensions of 5×10^3^ cells were seeded in six replicates into each well of 24-well cell culture plates (Corning, Inc.) in selective media and placed in Incucyte. Incucyte was programmed to image each well at 3-hour intervals. Data analysis was conducted using Incucyte 2011A software.

### Transwell migration and invasion assays

Matrigel inserts were hydrated for 2 hours in 500 uL serum-free DMEM then suspensions of 7.5×10^4^ cells were seeded in triplicate into each well of the 24-well plate format BD BioCoat Control and Matrigel Invasion Chambers (BD Biosciences) and incubated for 20 hours at 37°C in 5% CO_2_. Enriched media was used as chemoattractant. Membranes were subsequently fixed with methanol, stained with crystal violet, and cells present on the underside of the membrane were counted.

### Three-dimensional culture

Cultrex 3-D culture matrix (Trevigen, Inc.) was injected into 8-chamber slides and allowed to solidify at 37°C for 30 minutes. Cells were trypsinized and 5,000 counted then resuspended in 400 uL DMEM, 9%FBS, 5 ug/mLBlasticidin, and 2% Cultrex and incubated at 37°C with 5% CO_2_ for 3 days prior to microscopy.

### Mouse work: Orthotopic and tail vein injections

Female FVB/NJ mice from Jackson Laboratories were injected at 6–8 weeks of age. Two days prior to orthotopic injections, cells were placed in non-selective media. On the day of injection, 1×10^5^ cells were injected orthotopically into the fourth mammary fat pad of age-matched virgin females. After 30 days the mice were euthanized by intraperitoneal injection of 1 mL Tribromoethanol with subsequent cervical dislocation. Primary tumors were resected, weighed, and snap frozen in liquid nitrogen. Lungs were resected, surface metastases were counted; lungs were inflated with 10% nitrate-buffered formalin and sent for sectioning and staining. For tail vein injection, 7×10^5^ were injected into the lateral tail vein, mice were euthanized 22 days post-injection. For the CD8-antibody treatment study, orthotopic transplantation was conducted but mice were treated with 0.5 mg of either control rat IgG or rat monoclonal anti-CD8 IgG (Harlan Bioproducts, kindly provided by Dr. Lalage Wakefield) on days −4, −3, −2, +3, +10, +17, +24, and +30; cells were injected on day 0 and mice were euthanized on day 37. All procedures were performed under the Animal Safety Proposal (LCBG-004) and approved by the NCI-Bethesda Animal Care and Use Committee.

### Immunohistochemistry

Immunohistochemical staining was performed on LeicaBiosystems' Bond Autostainer on paraffin embedded tissue sections with biotinylated rabbit anti-Goat IgG; primary antibody, anti-V5 rabbit polyclonal antibody (Abcam Catalog #: ab95038); LeicaBiosystems Intense R Detection Kit. All histological analysis including paraffin embedding, sectioning, hematoxylin and eosin staining and immunohistochemistry were conducted by the Pathology/Histotechnology Laboratory, Laboratory Animal Sciences Program, SAIC, Frederick.

### IFN-γ ELISPOT

For IFN-γ production by T cells in either *Cadm1*-expressing or control tumor bearing mice: Single cell suspensions from the lymph nodes of tumor bearing mice were prepared. 1.6 million cells were loaded into each well of an IFN-γ ELISPOT plate, stimulated with anti-CD3 (0.5 ug/ml, eBioscience, San Diego, CA), and cultured overnight. The procedure was done according to recommendations from the manufacture (BD). Three to four mice for each experimental group, and triplicates for each sample were examined. The ELISPOT plate was scanned in ImmunoSpot (Cellular Technology Ltd. Shaker Heights, OH) and quantification was assessed using the CTL Scanning and CTL counting 4.0.

### Statistical analysis

Statistical analysis comparing two samples were conducted using the Mann-Whitney test on Prism Version 5.03 (GraphPad Software, La Jolla, CA). Multiple-comparison data was analyzed by Kruskal-Wallis test with post-hoc Conover-Inman correction for multiple analyses by R-script. Survival data was conducted with the Mantel-Cox test on Prism.

## Supporting Information

Figure S1 NZB and C58 chromosome 9 metastasis QTL.
(EPS)

Figure S2 Detection of synonymous SNP in *Cadm1* exon 2 (rs32721609).
(EPS)

Figure S3 Metastatic nodule cross-sectional area (um^2^) was measured on hematoxylin and eosin stained sections, no statistically significant difference was observed (Mvt-1 Vector n = 275, Mvt-1-Cadm1 n = 44, 6DT1 Vector n = 38, and 6DT1 Cadm1 n = 22 metastatic nodules).
(EPS)

Figure S4 No significant difference observed in the growth rate or cellular phenotype of *Cadm1* expressing Mvt-1 and 6DT1 cells relative to control.
(TIF)

Figure S5 Growth in three-dimensional culture. After growth Cultrex-containing rich media for 3 days, cells showed no difference in growth in three dimensional culture.
(EPS)

Figure S6 Effect of *Cadm1* expression on trans-well migration and invasion. *Cadm1*-expressing Mvt-1 (a and c) and 6DT1 (b and d) cells were assayed for serum-stimulated transwell migration through porous Polytetrafluoroethylene and invasion through matrigel.
(EPS)

Figure S7 Motility of *Cadm1* expressing Mvt-1 and 6DT1 cells relative to control. *Cadm1* expression had no effect on Mvt-1 cell motility but resulted in a minor reduction in motility of 6DT1 cells.
(TIF)

Figure S8 Flow cytometry demonstrating CD8^+^ T cell depletion in the peripheral blood of one representative sample from each group (a, b, c, d).
(EPS)

Figure S9 Protein-level *Cadm1* expression in Mvt-1 cell lines directly prior to injection into animals for *in vivo* studies in immune-competent (FVB), athymic (nude), and antibody treated mice.
(EPS)

Table S1 Breast cancer patient dataset details. n, number of patients per group; LN, lymph node; ER, estrogen receptor; IDC, invasive ductal carcinoma; TCGA, The Cancer Genome Atlas.
(EPS)

Table S2 Primer sequences.
(DOC)
