# Supplementary figures and images for: Cadm1 Is a Metastasis Susceptibility Gene That Suppresses Metastasis by Modifying Tumor Interaction with the Cell-Mediated Immunity
Source: PLoS Genet. 2012 Sep 20;8(9):e1002926. doi: 10.1371/journal.pgen.1002926 (PMC3447942; doi:10.1371/journal.pgen.1002926)

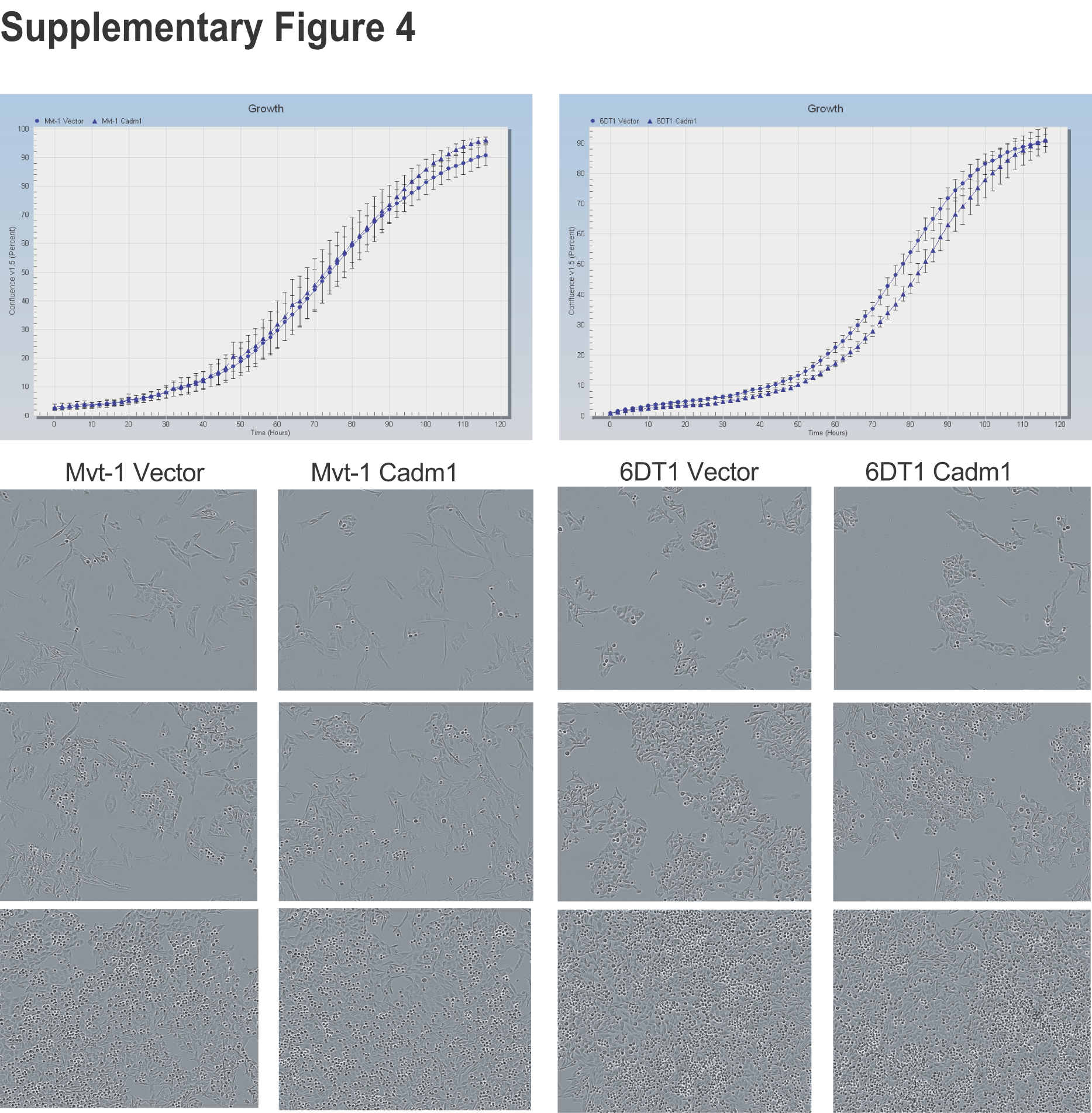

Supplement: Figure S4 — No significant difference observed in the growth rate or cellular phenotype of Cadm1 expressing Mvt-1 and 6DT1 cells relative to control. (TIF) [file pgen.1002926.s004.tif]

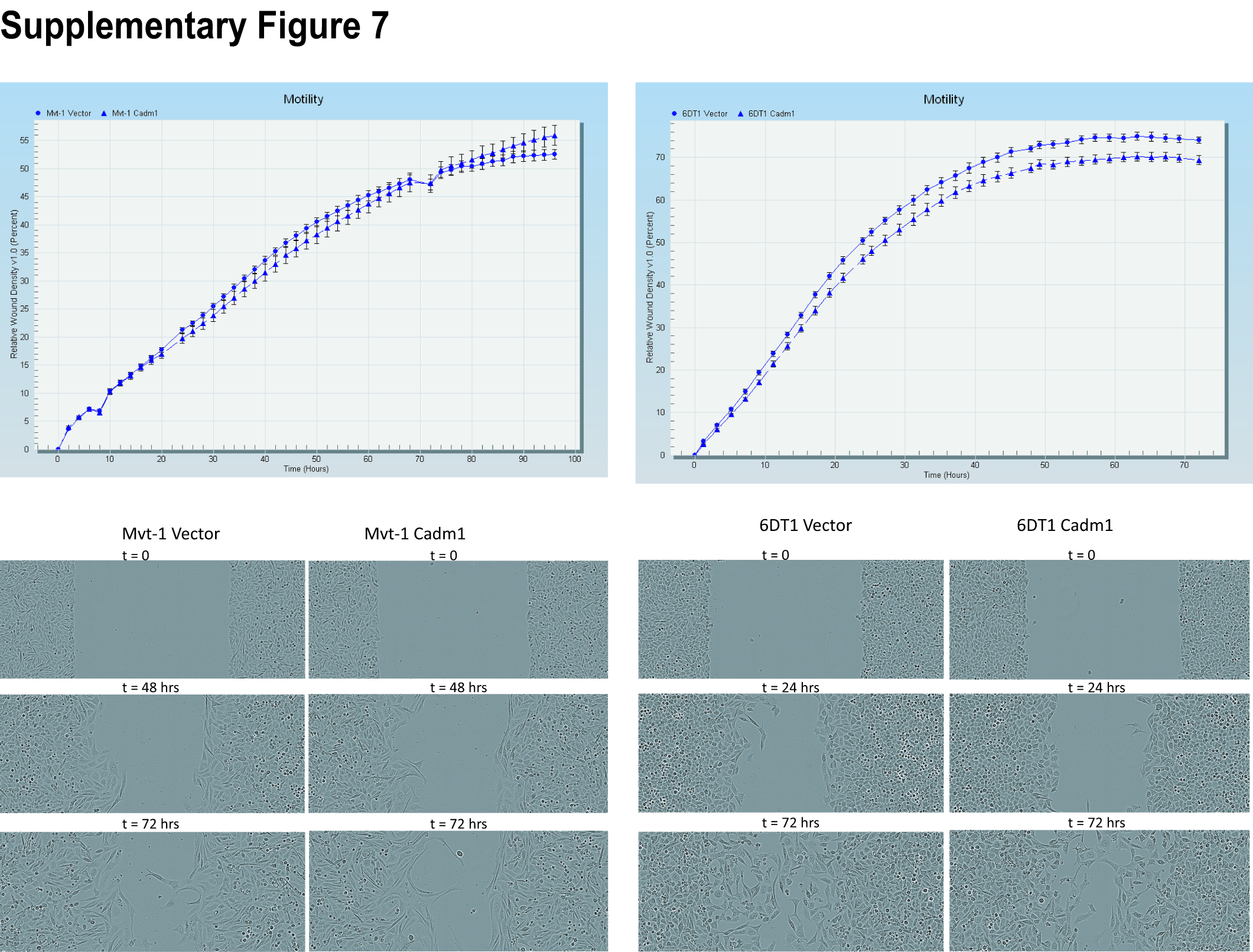

Supplement: Figure S7 — Motility of Cadm1 expressing Mvt-1 and 6DT1 cells relative to control. Cadm1 expression had no effect on Mvt-1 cell motility but resulted in a minor reduction in motility of 6DT1 cells. (TIF) [file pgen.1002926.s007.tif]
