# Supplementary material for: Cadm1 Is a Metastasis Susceptibility Gene That Suppresses Metastasis by Modifying Tumor Interaction with the Cell-Mediated Immunity
Source: PLoS Genet. 2012 Sep 20;8(9):e1002926. doi: 10.1371/journal.pgen.1002926 (PMC3447942; doi:10.1371/journal.pgen.1002926)
Supplement: Table S2 — Primer sequences. (DOC) [file pgen.1002926.s011.doc]

Exon sequencing primers:

| *Cadm1* | *Cadm1*_ex1F2 | AGGTGCCACGATTGGTCGCT |  |  |  |  |  |
| --- | --- | --- | --- | --- | --- | --- | --- |
|  | *Cadm1*_ex1R2 | GGGGGAAGGTTGTCATGGAA |  |  |  | | |
|  | *Cadm1*_ex2F | GTTTTGTAATGCGTTTCTCA | | |  | | |
|  | *Cadm1*_ex2R | GCAATCTCTTTATCAAGACA |  |  |  | | |
|  | *Cadm1*_ex3F | CATCTGACGTTTTTCCTTTGCCTA |  |  |  | | |
|  | *Cadm1*_ex3R | TGCTACTGGAAGCCATTTGG |  |  |  | | |
|  | *Cadm1*_ex4F | TGACACTGGTCCCTAAGGAA |  |  |  | | |
|  | *Cadm1*_ex4R | CAACTGTTTGTAATTTCCGC |  |  |  | | |
|  | *Cadm1*_ex5F | TGACGTTGCCATCTTGTTACCT |  |  |  | | |
|  | *Cadm1*_ex5R | CCTCGTACACCATACAGATT |  |  |  | | |
|  | *Cadm1*_ex6F | TGGCGGTGTGGGCTTTGTGA |  |  |  | | |
|  | *Cadm1*_ex6R | GCCAATGAGCTGTGAATGCA |  |  |  | | |
|  | *Cadm1*_ex7F | ACTGGATACAGATACCTTAGTGGG |  |  |  | | |
|  | *Cadm1*_ex7R | AAAGGCTTTGGGGTGAAGCC |  |  |  | | |
|  | *Cadm1*_ex8.2F | GCAACTAAGGCTTTAGGGATAGT |  |  |  | | |
|  | *Cadm1*_ex8.2R | CAGGGAACAGCTAGTCTTCTTTT | | |  | | |
|  | *Cadm1*_ex9F | TGGGGCCCTTGGTGTCTTGT | | |  | | |
|  | *Cadm1*_ex9R | CACCAAACTGCCTACTATAA |  |  |  | | |
|  | *Cadm1*_ex10F2 | ACGCCTTCTTCTCTGGTCATAA |  |  |  | | |
|  | *Cadm1*_ex10R2 | TTCCCAGTCTCGCATCTCTC | | |  | | |

**qRT-PCR primers:**

For measurement of total *Cadm1* RNA:

*Cadm1*-qPCR-For1 TTTGAAGGACAGCAGGTTTCAG

*Cadm1*-qPCR-Rev1 AGGACTGTGATGGTGGTGTAACTC

For measurement of exogenous *Cadm1* RNA:

*Cadm1*-qPCR-For3 GCCAGACATAAAGGTACATA

V5-Rev ACCGAGGAGAGGGTTAGGGAT

Normalizing control:

PPiB-F TTTGAAGGACAGCAGGTTTCAG

PPiB-R AGGACTGTGATGGTGGTGTAACTC
